# Intestinal Sclerostin Deficiency Links Gut Dysbiosis to Altered Serotonin Homeostasis in Axial Spondyloarthritis

**DOI:** 10.1007/s10753-025-02393-3

**Published:** 2026-01-10

**Authors:** Daniele Mauro, Anne-Sophie Bergot, Giuliana Guggino, Alessia Salzillo, Giulio Forte, Antonio Ciancio, Aroldo Rizzo, Stefania Raimondo, Luca Lentini, Saviana Gandolfo, Soohyun P. Kim, Chi Wong, Barbara De Marino, Simon Milling, Riccardo Alessandro, Iacopo Panarese, Ryan C. Riddle, Mario M. Zaiss, Dennis McGonagle, Ranjeny Thomas, Francesco Ciccia

**Affiliations:** 1 Dipartimento di Medicina di Precisione, Università della Campania “Luigi Vanvitelli”, Naples, Italy; 2 The University of Queensland Diamantina Institute, Translational Research Institute, Brisbane, QLD Australia; 3 Dipartimento PROMISE, University of Palermo, Palermo, Italy; 4 Azienda Ospedaliera Ospedali Riuniti Villa Sofia-Cervello, Palermo, Italy; 5 Dipartimento di Biomedicina, Neuroscienze e Diagnostica Avanzata (Bi.N.D), Università di Palermo, Palermo, Italy; 6 Rheumatology Unit, Ospedale del Mare, Naples, Italy; 7 Department of Orthopedics, University of Maryland School of Medicine, Baltimore, MD USA; 8 School of Infection and Immunity, University of Glasgow, Glasgow, UK; 9 Pathology Unit, Dipartimento di Salute Mentale, Fisica e Medicina Preventiva, Università degli Studi della Campania ’Luigi Vanvitelli’, Naples, Italy; 10 Department of Internal Medicine 3, Rheumatology and Immunology, Friedrich-Alexander- University Erlangen-Nürnberg (FAU) and Universitätsklinikum Erlangen, Erlangen, Germany; 11 Deutsches Zentrum Immuntherapie (DZI) FAU Erlangen-Nürnberg and Universitätsklinikum Erlangen, Erlangen, Germany; 12 Leeds Biomedical Research Centre, University of Leeds, Leeds, UK

**Keywords:** Sclerostin, Axial spondyloarthritis, Serotonin, Dysbiosis

## Abstract

**Background:**

Sclerostin regulates bone formation via Wnt/β-catenin signaling inhibition and contributes to intestinal epithelial homeostasis. Circulating sclerostin levels are reduced in axial spondyloarthritis (axSpA) and correlate with structural damage. LRP5, a receptor inhibited by sclerostin, also controls bone formation by regulating gut-derived serotonin synthesis, indicating a hormonal link between the intestine and bone. We hypothesized that gut dysbiosis-dependent downregulation of sclerostin alters intestinal serotonin production, contributing to disease-specific gut-bone signaling in axSpA.

**Methods:**

We quantified sclerostin and the serotonin-synthesizing enzyme TPH1 by qRT-PCR, and assessed serotonin protein levels by immunohistochemistry in ileal biopsies from treatment-naïve axSpA patients (*n* = 25) and healthy controls (*n* = 20), alongside measurement of circulating serotonin in peripheral blood platelets. We evaluated TPH1 expression in BON-1 cells following sclerostin and WNT3a treatment. Findings were validated in HLA-B27 transgenic rats, SKG mice, and Sost⁻/⁻ mice. Serotonin receptor expression in spinal entheseal cells was analyzed by RT-PCR, and LPS-induced HTR2B modulation was examined.

**Results:**

In healthy controls, sclerostin modulated TPH1 expression and serotonin synthesis in enterochromaffin cells. In axSpA patients, intestinal sclerostin downregulation coincided with increased numbers of serotonin-positive enterochromaffin cells and elevated platelet serotonin levels. Broad-spectrum antibiotics restored intestinal sclerostin expression and normalized serotonin production in HLA-B27 transgenic rats. Sost⁻/⁻ mice exhibited increased intestinal Tph1 expression, while SKG mice showed reduced sclerostin and elevated Tph1 following curdlan-induced colitis—an effect dependent on the presence of intestinal microbiota. Human spinal entheses expressed HTR1B, HTR2A, and HTR2B, with LPS selectively inducing HTR2B expression.

**Conclusions:**

We identify a gut microbiota-dependent sclerostin-serotonin axis that regulates serotonin production and may contribute to gut-bone pathology in axSpA. These findings reveal novel mechanisms linking gut dysbiosis to bone disease and suggest potential therapeutic targets within the gut-bone-immune axis.

**Supplementary Information:**

The online version contains supplementary material available at 10.1007/s10753-025-02393-3.

## Introduction

Sclerostin is an osteocyte-derived glycoprotein that binds LRP5, inhibiting Wnt/β-catenin signaling and suppressing osteoblast differentiation and proliferation, thereby regulating bone mass [1]. Wnt/β-catenin signaling also controls intestinal epithelial stem cell proliferation and influences mucosal inflammation and repair [2]. In the gut, LRP5 represses expression of tryptophan hydroxylase 1 (TPH1), the rate-limiting enzyme for serotonin biosynthesis in enterochromaffin cells [3]. In LRP5‐deficient mice, reducing serotonin levels restores bone formation and mass, highlighting a physiologic neuroendocrine serotonin‐dependent gut–bone axis [4].

Axial spondyloarthritis (AxSpA) is a chronic inflammatory disease of the spine and sacroiliac joints characterized by innate immune activation, chronic inflammation, and pathologic new bone formation [5–7]. In AxSpA patients, sclerostin expression is impaired [8], with lower levels correlating with more severe structural damage [9] and predicting axSpA in inflammatory bowel disease patients [10]. Furthermore, AxSpA patients exhibit elevated serum levels of sclerostin–IgG immune complexes, including IgG reactive to the SOST-S146 peptide, which shares homology with a filamentous bacterial glycotransferase peptide [9]. Solid evidence demonstrates intestinal dysbiosis in AxSpA, emphasizing crosstalk between an altered microbiome and the innate immune system in genetically predisposed individuals; this interaction modulates innate and adaptive immune responses and supports an AxSpA gut–bone axis [11–14].

Despite these insights, the regulation of intestinal sclerostin production and its impact on serotonin homeostasis and innate immunity in AxSpA remain undefined. We hypothesized that gut microbial dysbiosis downregulates intestinal sclerostin, leading to increased TPH1 expression, elevated serotonin in the gut and circulation, which may contribute to the gut-bone axis. To test this, we investigated sclerostin production in healthy and AxSpA conditions, assessed the role of intestinal bacteria and their metabolites in modulating sclerostin expression, and employed HLA-B27 transgenic rats, SKG, and SOST^−/−^ mice to dissect how gut dysbiosis, arthritis, and sclerostin insufficiency influence serotonin production.

## Materials and Methods

### Participants Recruitment

We obtained blood and ileal biopsies from 25 treatment-naïve axial spondyloarthritis (AxSpA) patients and 20 healthy controls (HC) after informed consent, under protocols approved by the Institutional Review Boards of the University of Palermo (5/201416042014) and the University of Campania Luigi Vanvitelli (2021.001336). AxSpA patients fulfilled the Assessment of Spondyloarthritis International Society (ASAS) classification criteria and were recruited at University Hospital “Luigi Vanvitelli” (Naples) and University Hospital “Paolo Giaccone” (Palermo). Patient characteristics are detailed in Supplementary Table S1. We also included six patients with active Crohn’s disease, defined by a Crohn’s Disease Activity Index (CDAI) > 150, as an additional disease control.

### Histology, Immunohistochemistry, and Immunofluorescence

We obtained ileal mucosal biopsies from treatment-naïve AxSpA patients and healthy controls and conducted immunohistochemical staining for sclerostin, serotonin, and TPH1 on paraffin-embedded ileal sections and analyzed them by immunohistochemistry (IHC), following established methods [7]. Primary and secondary antibodies are listed in Supplementary Table 2. Two independent researchers (FC and AR) manually counted positive cells in photomicrographs from three randomly selected high-power fields (40× magnification) per section, acquired on a Leica DM2000 microscope with a Leica DFC320 camera. Intra- and inter-rater reliability, assessed by Cohen’s κ, were 0.80 and 0.76, respectively.

### Cell Line Experiments

The human neuroendocrine tumor cell line BON-1 was cultured in DMEM/F12 medium supplemented with 10% fetal bovine serum, 1% penicillin-streptomycin, and 1% L-glutamine at 37 °C in 5% CO₂. For stimulation assays, cells were seeded on standard plates or Nunc Lab-Tek II 8-well chamber slides and treated for 24–48 h with recombinant human sclerostin (10 nM, Abcam), WNT3a agonist (2.5 nM, Abcam), both, or left untreated. For immunofluorescence, after 48 h, cells were fixed in methanol at − 20 °C, stained with rabbit anti-human TPH1 (Abcam) or serotonin (Abcam) and Alexa Fluor 555-conjugated secondary antibody (Jackson Laboratories), counterstained with DAPI, and imaged on a Zeiss Axiotome microscope using identical settings.

Supernatants were collected at each time point, centrifuged at 1500 rpm for 5 min at 4 °C, and analyzed undiluted for serotonin using a Human Serotonin ELISA Kit (ab133053, Abcam) according to the manufacturer’s protocol. Absorbance was measured at 405 nm with reference correction (570–590 nm) on a TECAN reader.

For gene expression, total RNA was extracted from BON-1 cells after 24 h of treatment using TRIzol (Sigma-Aldrich), and qRT-PCR was performed.

For Western blot, BON-1 cells were lysed in RIPA buffer containing protease and phosphatase inhibitors after 48 h. SDS-PAGE separated equal amounts of protein, transferred to PVDF membranes, and probed with anti-TPH1 (Abcam) and HRP-conjugated secondary antibodies. Detection was by enhanced chemiluminescence (Thermo Fisher) and imaging (ChemiDoc, Bio-Rad); GAPDH served as loading control.

### Serotonin Quantification in Plasma and Platelets

Plasma was tested with the Human Serotonin ELISA Kit (ab133053, Abcam). To quantify platelet serotonin, blood was collected in citrate tubes from five healthy controls and five patients with axSpA. Platelet serotonin was measured on freshly collected samples from a subset of age- and sex-comparable AxSpA patients and HC, excluding known confounders such as ongoing treatment, medication use, or smoking, and processed immediately in parallel to minimize variability. Briefly, platelets were isolated by centrifugation, washed in DPBS, and smeared onto glass slides. After fixation in 4% paraformaldehyde, slides were blocked (Abcam), stained with rabbit anti-human serotonin (Abcam) and Alexa Fluor 488 488-conjugated secondary antibody (Jackson Laboratories), and counterstained with DAPI. The absence of DAPI staining confirmed the purity of the platelets. Images were acquired using a Zeiss Axiotome microscope with identical exposure settings, and the mean fluorescence intensity per platelet was quantified in ImageJ by defining regions of interest at the single-cell level.

### HLA-B27 Transgenic Rats and SKG Mouse Models

We used HLA-B2705 transgenic (B27-TG) rats (line 33 − 3; F344/NTac-Tg[HLA-B2705, β2M]; Taconic) backcrossed for ≥ 10 generations onto the PVG/OlaHsd background (PVG/OlaHsd; Harlan), as described [15]. We collected serum samples before and after broad-spectrum antibiotic treatment as detailed before [14].

Female BALB/c and SKG (ZAP-70^W163C) mice, kindly provided by S. Sakaguchi (University of Kyoto), were bred and maintained under specific pathogen-free or germ-free conditions at the University of Queensland Translational Research Institute Animal Facility. Mice were kept on a 12-h light/12-h dark cycle with ad libitum access to food and water and used at 8–12 weeks of age under protocols approved by the University of Queensland Animal Ethics Committee. We treated SKG mice with either vehicle or intraperitoneal curdlan (1,3-β-glucan; 15 mg/mL in saline). Seven days post-treatment, we harvested tissues for sclerostin immunostaining on serial 5-µm paraffin sections and extracted RNA from RNAlater-stored samples for RT-PCR analysis of *Tph1* and expression of related genes.

### Generation and Maintenance of Sost^-/-^ Mice

We obtained Sost–/– mice (Sost^tm1(KOMP)Vlcg) from the KOMP Repository (www.komp.org). The Velocigene-targeted allele was generated as described previously [16]. Founder animals were produced by fertilizing wild-type C57BL/6 oocytes with sperm from Sost^+⁄–^ males. Sost^–/–^ and Sost^+/+^ littermate controls were then derived by intercrossing heterozygotes and maintained on a C57BL/6 background [17].

All mice were housed in a specific-pathogen-free facility on ventilated racks under a 14-h light/10-h dark cycle, with ad libitum access to water and a standard rodent diet (Extruded Global Rodent Diet, Harlan Laboratories).

### RNA Extraction and Quantitative Real-Time PCR Analysis

Total RNA was isolated using the Illustra RNAspin Mini Isolation Kit (GE Healthcare, UK) and reverse-transcribed into cDNA with the High-Capacity cDNA Reverse Transcription Kit (Applied Biosystems). Quantitative real-time PCR (qRT-PCR) was performed using SYBR Green Master Mix on the StepOne Real-Time PCR System (Applied Biosystems), comparing gene expression between control subjects and AxSpA patients. GAPDH and 18 S rRNA were evaluated as endogenous controls and yielded comparable results; GAPDH was selected for data normalization. Relative gene expression was calculated by the ΔΔCt method and is presented as fold induction relative to control levels.

### Spinal Enthesis Cell Isolation and Serotonin Receptor qPCR

The investigation was approved by the North West–Greater Manchester West Research Ethics Committee (REC: 16/NW/0797). All patients gave written informed consent, in accordance with the Declaration of Helsinki. Human peri-entheseal bone (PEB) from the spinous process and entheseal soft tissue (EST) from the interspinous ligament were collected during spinal surgery from donors without inflammatory disease cells as previously described [18].

Entheseal tissue was mechanically dissected and digested. The resulting cell suspension was plated at 0.5 × 10^6^ cells/well in DMEM high glucose (Gibco) supplemented with 10% fetal calf serum (Thermo Fisher), 1% penicillin/streptomycin. Cells were cultured at 37 °C, 5% CO₂ for 24 h and then stimulated in duplicate under two conditions: untreated control or lipopolysaccharide (LPS, 100 ng/mL; Sigma-Aldrich).

RNA was extracted with TRIzol (Invitrogen), reverse-transcribed using the High-Capacity cDNA Reverse Transcription Kit (Applied Biosystems), and analyzed by quantitative real-time PCR with TaqMan assays for HTR2A, HTR2B, HTR2C, and HTR1B, using HPRT1 as the reference gene. Relative expression levels were calculated by the ΔΔCt method.

### Data Analysis

The statistical analysis was conducted in GraphPad Prism 5. Differences in quantitative variables were analyzed using the Mann-Whitney test. Differences across multiple groups were tested by one-way ANOVA followed by Tukey’s multiple comparison test. *P*-values < 0.05 were considered statistically significant.

### Data Sharing

Datasets are available on reasonable request to the corresponding author.

## Results

### Sclerostin Modulatory Effect on Serotonin Production Via TPH1 in Enterochromaffin Cells

Previous research has established the WNT coreceptor Lrp5 as a regulator of *Tph1* expression in mice, highlighting its pivotal role in serotonin production by enterochromaffin cells. Given that sclerostin functions as a WNT signaling inhibitor, we aimed to elucidate sclerostin’s impact on TPH1 expression and subsequent serotonin production in a human enterochromaffin cell line (BON-1).

**Fig. 1:**
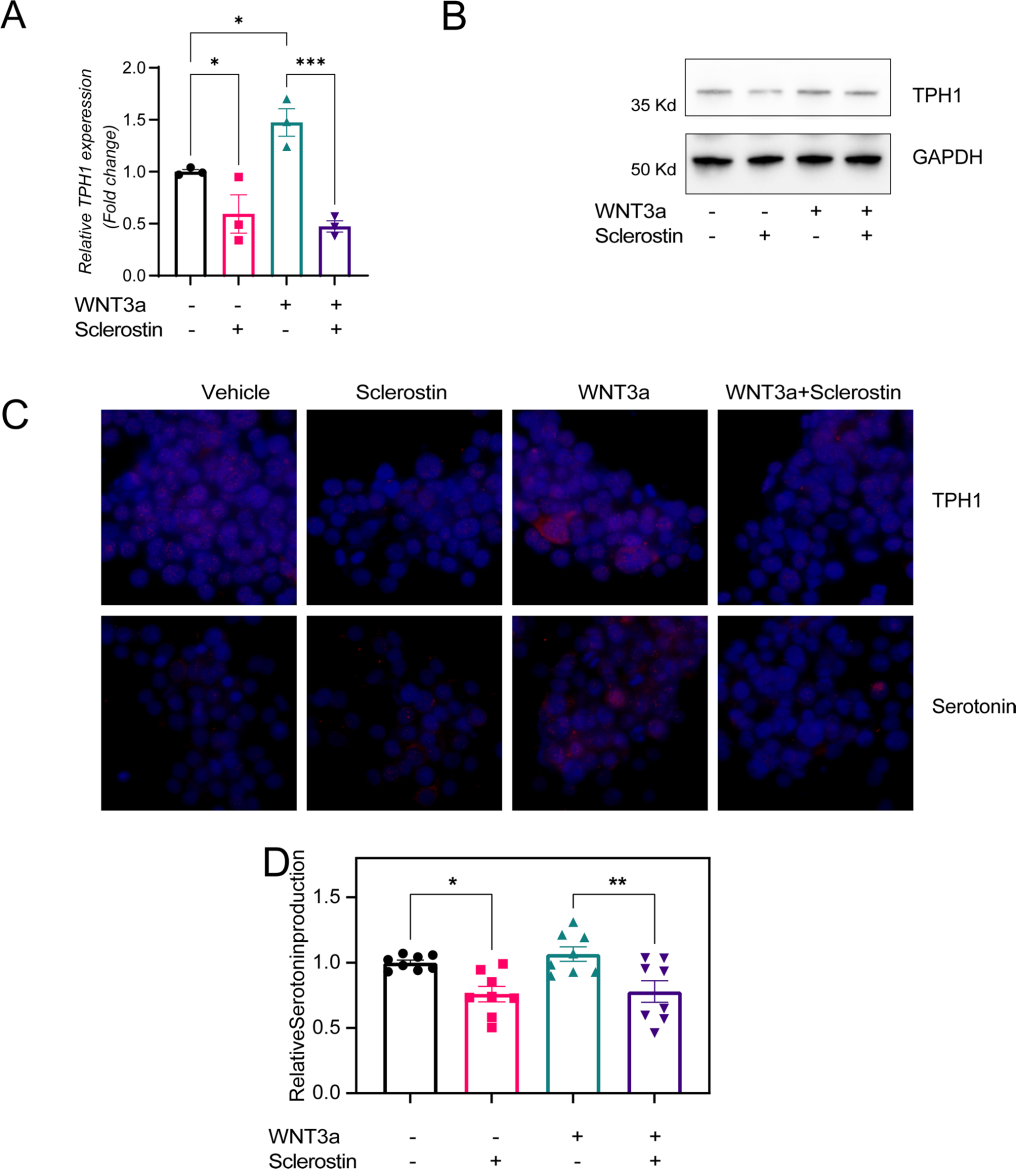
Sclerostin modulates TPH1 expression and Serotonin production in enterochromaffin cells. BON-1 cells underwent treatments with human recombinant sclerostin (10 nM), recombinant human WNT3a (2.5 nM), their combination, or left untreated (control) for 24 h, after which RNA and proteins were extracted for analysis. (**A**) shows the quantification of *TPH1* mRNA levels by qRT-PCR. (**B**) presents TPH1 protein levels assessed by Western blot, with GAPDH serving as the loading control. For immunofluorescence analysis, BON-1 cells were cultured on microscope slides, treated as above for 48 h, and then stained for intracellular TPH1 and serotonin detection (Nuclei are stained with DAPI in blue, and TPH1 or serotonin in red); a representative image from three replicates is shown. Serotonin levels in the supernatant were measured by competitive ELISA, with results expressed as fold change relative to the untreated control for each independent experiment. Statistical significance was determined using ANOVA, with * indicating *p* < 0.05, and ** indicating *p* < 0.01

Consistent with expectations, WNT3a stimulation in vitro significantly upregulated TPH1 expression, the rate-limiting enzyme in serotonin synthesis. Notably, sclerostin treatment effectively diminished *TPH1* expression in both basal and WNT3a-activated BON-1 cells (Fig. 1A). This suppressive effect of sclerostin on *TPH1* expression was further validated at the protein level through Western blot analysis, revealing a decrease in intracellular TPH1 levels under both conditions (Fig. 1B). Immunofluorescence assays on adherent BON1 cells corroborated these findings, showing that WNT3a-mediated increases in TPH1 expression were significantly attenuated by sclerostin treatment (Fig. 1C).

Moreover, the functional consequence of TPH1 inhibition by sclerostin was substantiated by the observed reduction in serotonin secretion into the culture supernatant, as quantified by ELISA (Fig. 1D). These results collectively underscore sclerostin’s regulatory influence on serotonin production, mediated through its inhibitory action on *TPH1* expression in enterochromaffin cells.

### Sclerostin Is Produced in the Healthy Gut and Downregulated in axSpA

**Fig. 2:**
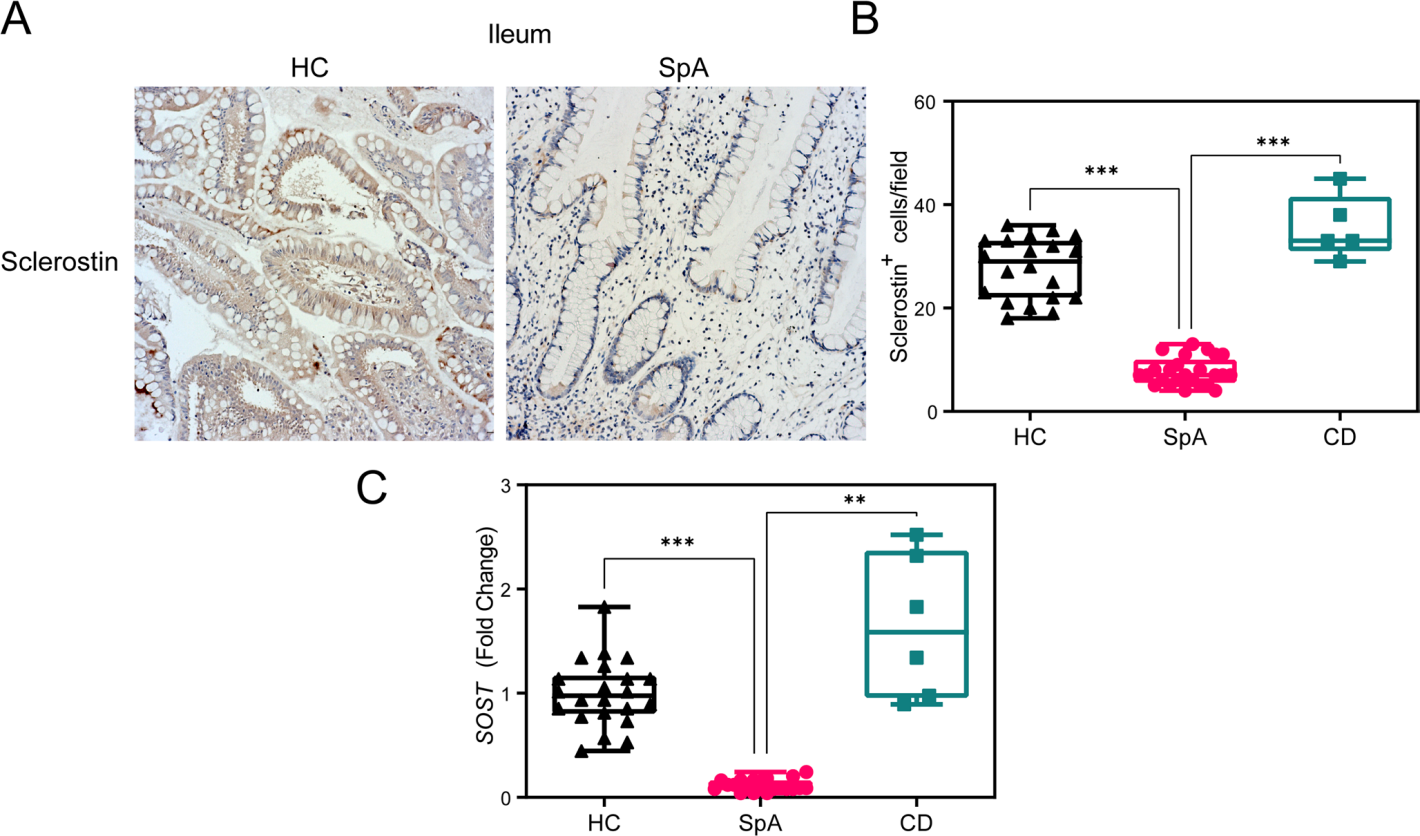
Intestinal epithelial sclerostin production is downregulated in SpA. (**A**) Representative IHC images of ileal biopsies obtained from HC and SpA stained for Sclerostin and counterstained with hematoxylin (*n* = 20 and 25, respectively). (**B**) Quantification of the Sclerostin^+^ cells per high magnification field (40 x) in ileal tissue obtained from HC (*n* = 20), SpA patients (*n* = 25), and Crohn’s Disease patients (CD) (*n* = 5). (**C**) quantification of *SOST* mRNA levels by qRT-PCR in SpA (*n* = 22), HC (*n* = 20), and CD (*n* = 6). Statistical significance was determined using ANOVA, with * indicating *p* < 0.05, and ** indicating *p* < 0.01. *** indicating *p* < 0.001

Having shown that Sclerostin may play a role in the modulation of serotonin production in enterochromaffin cells, we investigated whether sclerostin is physiologically expressed in human intestinal tissue and whether reduced levels may be associated with axSpA also in the intestinal tissue, as previously demonstrated in other sites [8, 10, 19, 20] We observed basal sclerostin (*SOST* mRNA) expression in the normal intestine, which is preserved in the inflamed gut of patients with Crohn’s disease (CD). In contrast, axSpA was associated with a significant downregulation of sclerostin expression (Fig. 2). This observation was evident both at the protein level (Fig. 2A-B) and at the mRNA level (Fig. 2C).

### Sclerostin Downregulation and Increased Serotonin Production Are Linked To Dysbiosis and Colitis

The observed deficiency of sclerostin SpA is well documented across multiple independent studies in other tissues [8, 10, 19, 20]. Prompted by this, we investigated the underlying causes of sclerostin deficiency noted in the ileum of SpA patients. Given the association between intestinal dysbiosis, colitis, and SpA [13, 21], we employed SpA animal models to explore whether changes in the intestinal microbiota and the induction of colitis play crucial roles in suppressing sclerostin production and promoting serotonin production.

**Fig. 3:**
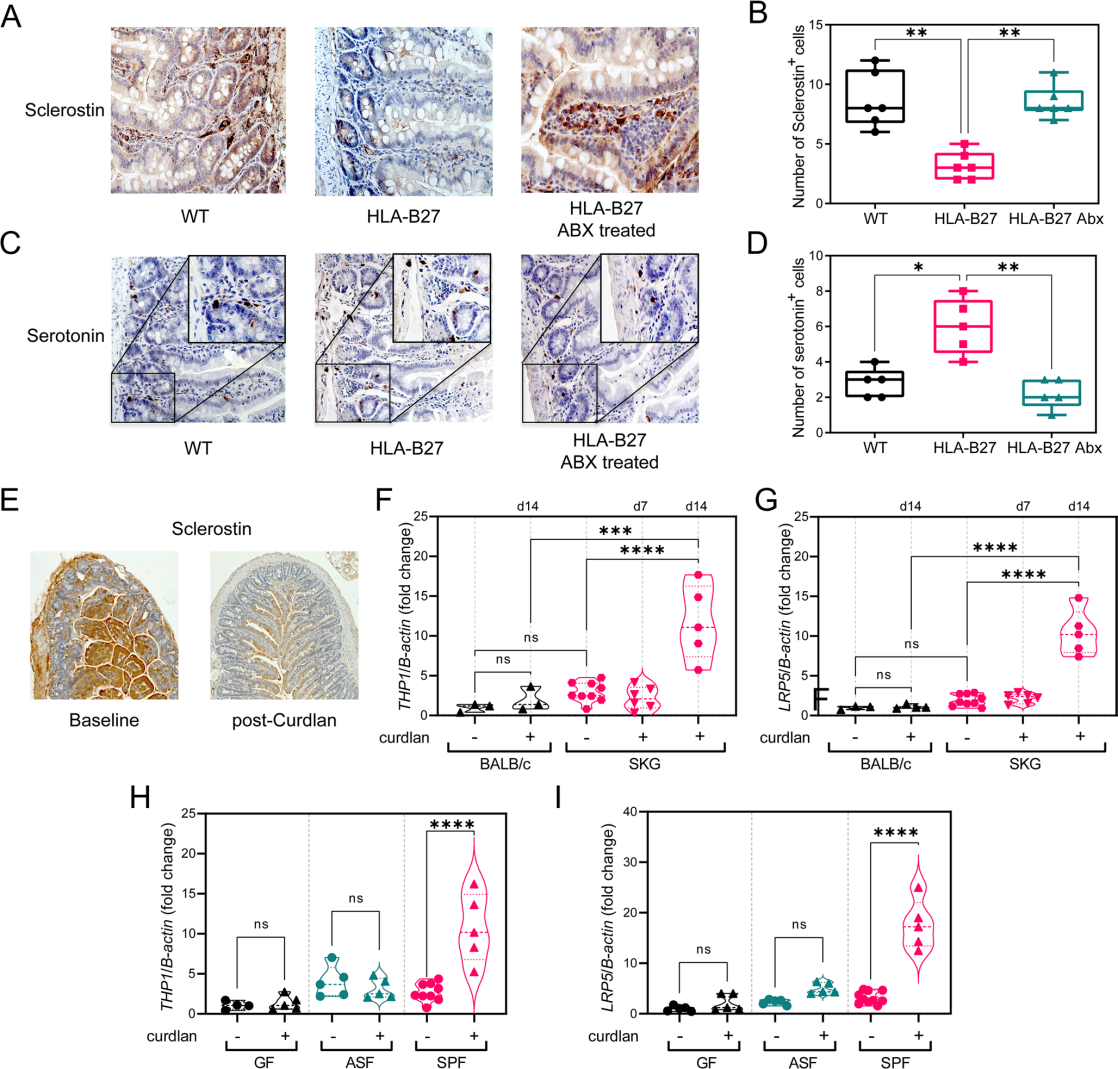
Impact of Microbiota and Ileitis on Sclerostin and Serotonin Regulation in SpA Models. Representative immunohistochemical images of intestinal tissue from wild-type (WT), HLA-B27 transgenic rats (HLA-B27), and HLA-B27 rats treated with antibiotics (ABX) showing sclerostin staining (**A**), with quantification presented in (**B**). Similarly, serotonin staining is displayed for the same groups (**C**), with corresponding quantification in (**D**). In (**E**) representative images of the intestine of untreated and curdlan i.p. treated SKG mice stained for sclerostin. mRNA expressions of *Tph1* and *Lrp5* in BALB/c and SKG mice at baseline and after 7 or 14 days of curdlan treatment are shown in (**F** and **G**). (**H**) and (**I**) shows *Tph1* and *Lrp5* expression in SKG mice kept under various microbial conditions: germ-free (GF), Altered Schaedler Flora (ASF), or specific pathogen-free (SPF), with or without curdlan treatment. Data, presented as mean ± SD for five replicates, were analysed using ANOVA, with statistical significance indicated as ns (*p* > 0.05), * (*p* < 0.05), ** (*p* < 0.01), *** (*p* < 0.001), **** (*p* < 0.0001)

In the HLA-B27 transgenic rat model, we noted a significant reduction in sclerostin levels compared to wild-type counterparts. This reduction appeared closely linked to the presence of microbiota, as antibiotic treatment effectively restored sclerostin levels to those observed in wild-type animals (Fig. 3A-B). Correspondingly, an inverse relationship was observed for serotonin, whose intestinal production increased in HLA-B27 transgenic rats relative to wild-type animals but normalized following antibiotic intervention (Fig. 3C-D).

Extending our investigation to the SKG mouse model, where arthritis and colitis are induced by intraperitoneal administration of curdlan, we sought to examine the consequent effects on sclerostin and *TPH1* expression. Consistent with observations in SpA patients and HLA-B27 transgenic rats, curdlan administration in SKG mice resulted in decreased intestinal sclerostin levels after 14 days (Fig. 3E) and a marked increase in *TPH1* mRNA expression compared to baseline conditions (Fig. 3F). Notably, these changes were specific to SKG mice, as wild-type BALB/c mice did not exhibit alterations in sclerostin or *TPH1* levels, neither at baseline nor following curdlan treatment. A corresponding modification in *LRP5* expression, the cognate receptor for sclerostin, paralleled the changes in sclerostin and *TPH1* mRNA levels (Fig. 3G**)**, reinforcing the interconnectedness of these pathways in the gut environment.

The involvement of the microbiota in these regulatory mechanisms was further substantiated. SKG mice maintained under germ-free conditions or colonized with Altered Schaedler Flora (ASF) did not show *TPH1* and *LRP5* upregulation following curdlan treatment (Fig. 3H-I**)**, highlighting the essential role of microbiota in this process.

### Sclerostin/Tph1 Axis in the Gut of SOST^-/-^ Mice

To investigate whether the increase in TPH1 and serotonin was caused by SOST deficiency, we then investigated the expression of intestinal *Tph1* in SOST^−/−^ mice.

SOST^−/−^ mice exhibit a high bone mass phenotype, characterized by significant increases in bone mineral density (BMD), bone volume, bone formation rates, and bone strength, as documented in prior studies [15, 22].

**Fig. 4:**
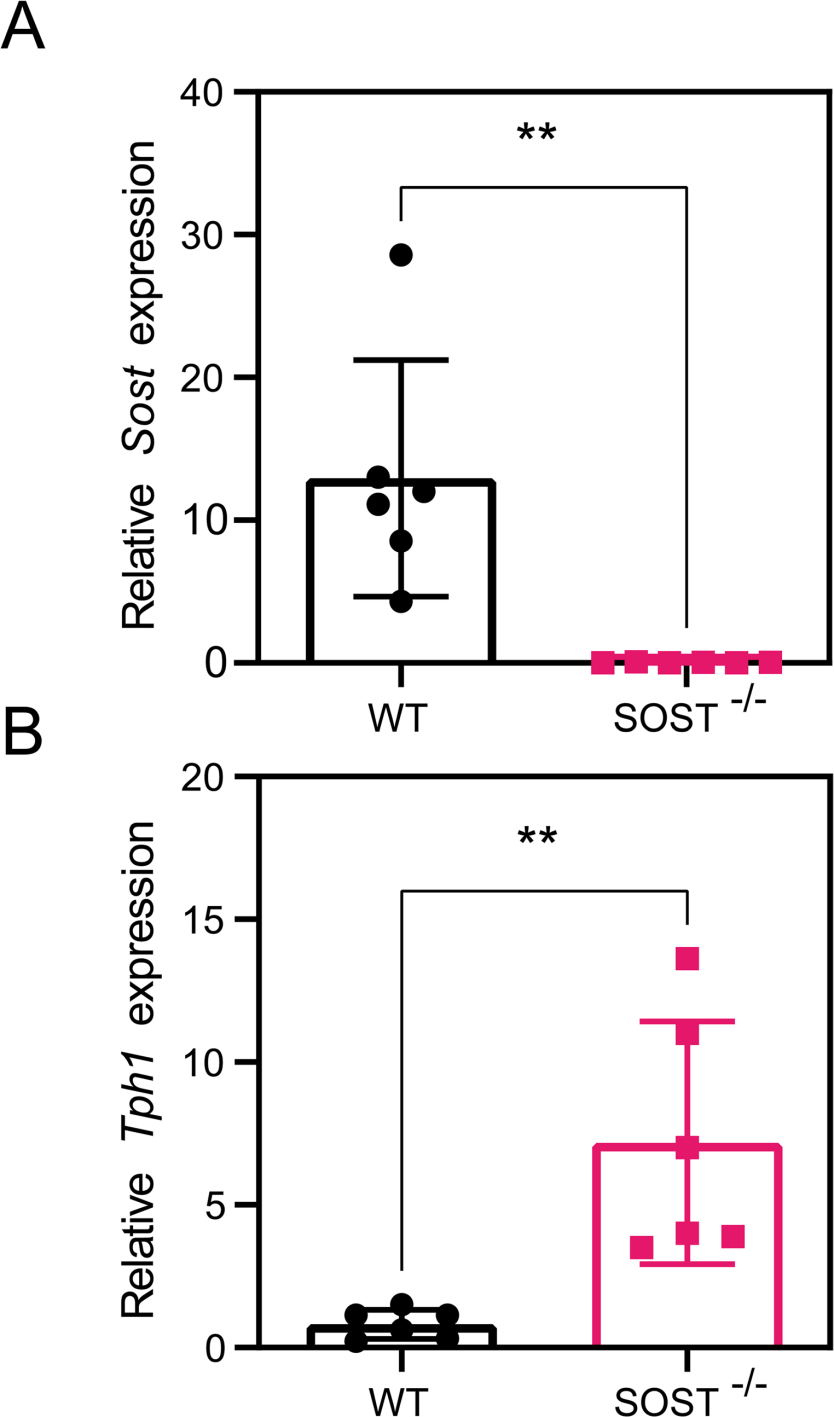
Expression of Tph1 in the gut of *SOST*^*-/-*^ mice (**A**) RT-PCR analysis of Sost mRNA levels in the intestine of wild-type and SOST-/- mice. (**B**) Corresponding RT-PCR analysis of Tph1 mRNA expression in the gut of wild-type and SOST-/- mice. Data, presented as mean ± SD for five replicates, with statistical significance indicated as ns (*p* > 0.05), ** (*p* < 0.01)

In wild-type mice, basal levels of intestinal *Sost* expression were detected via RT-PCR. In contrast, this expression was completely absent in SOST^−/−^ mice as expected (Fig. 4A). This absence of sclerostin correlated with a significant increase in *Tph1* expression in the gut of SOST^−/−^ mice (Fig. 4B). These findings confirm the regulatory role for sclerostin in serotonin biosynthesis pathways in the gut.

### In axSpA, Low Intestinal Levels of Sclerostin Are Associated with an Increase in Platelet Serotonin

Given our finding that sclerostin production is reduced in the gut of axSpA patients and that sclerostin regulates serotonin synthesis, we assessed serotonin levels in intestinal tissue and circulating platelets from axSpA patients and healthy controls. Platelets act as the principal reservoir of circulating serotonin by storing the monoamine produced and released into the bloodstream by ECs, thus modulating its peripheral availability [23].

**Fig. 5:**
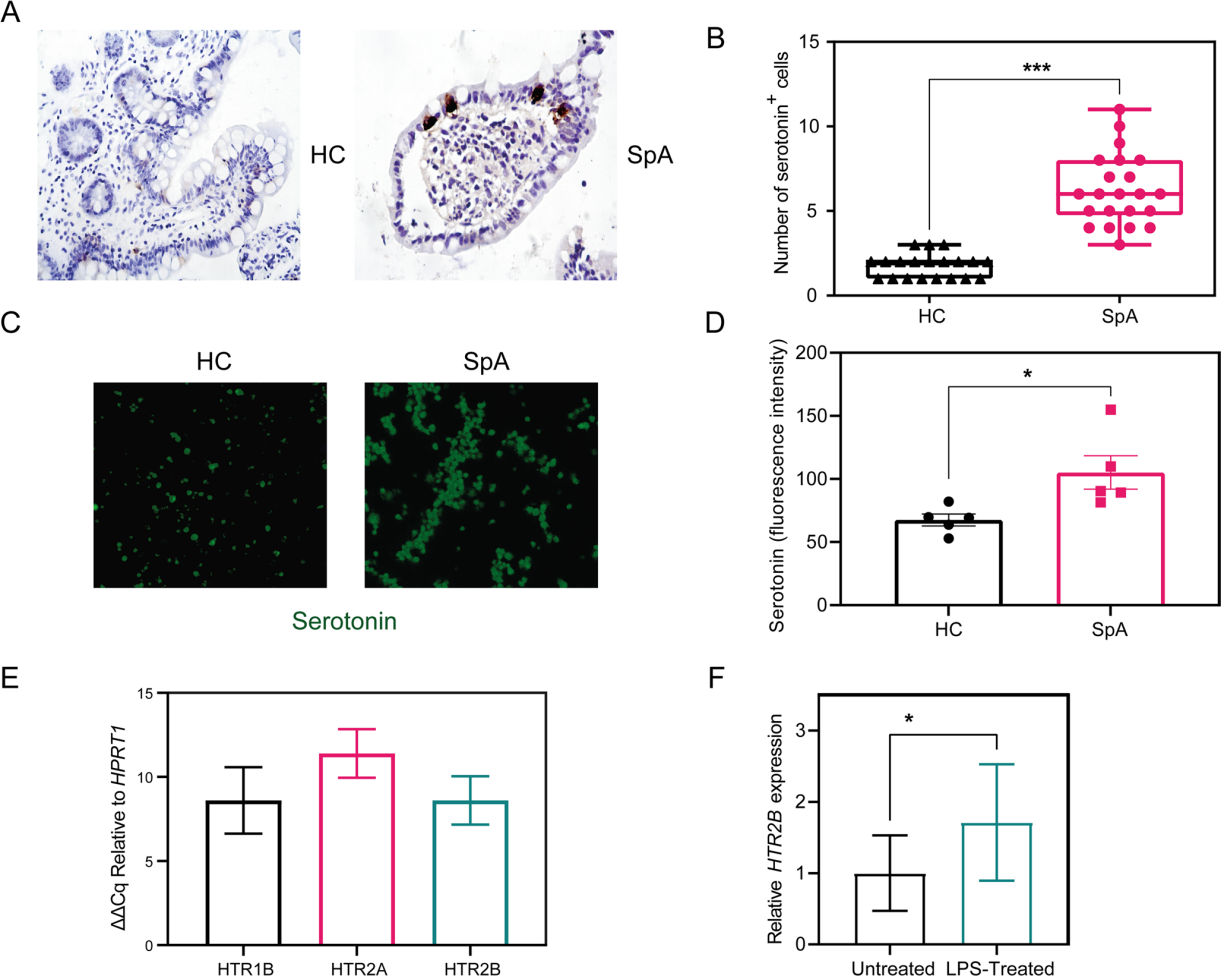
Serotonin levels in axSpA and serotonin receptor expression in spinal enthuses. (**A**–**B**) Immunohistochemical analysis of serotonin in ileal mucosa from healthy controls (HC) and axial spondyloarthritis (AxSpA) patients. (**A**) Representative images of serotonin-positive enterochromaffin cells in HC (left) and AxSpA (right). (**B**) Quantification of serotonin-positive cells per high-power field (40×). (**C**-**D**) Serotonin content in circulating platelets isolated from HC (*n* = 5) and AxSpA patients (*n* = 5), quantified by mean fluorescence intensity after Alexa Fluor 488 immunostaining. (**E**) RT-PCR analysis of serotonin receptor mRNA (*HTR1B*, *HTR2A*, *HTR2B*) in spinal entheseal cells from healthy donors (*n* = 8). (**F**) RT-PCR analysis of serotonin receptor *HTR2B* in cultured entheseal cells before and after LPS treatment (100 ng/mL). Data are presented as mean ± SEM; *p < 0.05, **p < 0.01 versus control

While no significant differences were observed using ELISA (data not shown), we found that reduced intestinal sclerostin in axSpA patients coincided with a higher number of serotonin-positive enterochromaffin cells compared to controls (Fig. 5A–B**).** In parallel, the intracellular serotonin content in circulating platelets was significantly increased in axSpA patients compared to controls (Fig. 5C-D). These findings support the existence of a gut-derived sclerostin–serotonin axis in axSpA.

Since serotonin can exert divergent effects on bone remodeling depending on the engagement of different receptor subtypes, we next examined serotonin receptor expression in spinal entheseal cells. Expression of *HTR1B*, *HTR2A*, and *HTR2B* was detected in healthy cells (Fig. 5E), while *HTR2C* was not detectable (data not shown). Upon in vitro stimulation with LPS, *HTR2B* expression was selectively upregulated in cultured entheseal stromal cells **(**Fig. 5E). Notably, HTR2B has been previously reported to exert anabolic effects on bone, suggesting that its induction may be relevant to serotonin-mediated signaling at the enthesis.”

## Discussion

Sclerostin is best known as an inhibitor of the Wnt/β-catenin pathway in bone, acting via LRP5/6 to suppress osteoblast differentiation [24–26]. Although initially described as osteocyte-specific, sclerostin is also produced by intestinal epithelial cells, and its receptor LRP5 is expressed in multiple tissues, suggesting a broader role in tissue homeostasis and immune regulation [17, 25–27].

Here, we demonstrate that sclerostin is also expressed by intestinal epithelial cells and gut-associated lymphoid tissue, where it contributes to immune regulation and mucosal homeostasis, which aligns with prior models in which IEC-derived signals modulate lymphoid structure development [28]. Our data show that sclerostin expression is markedly reduced in the gut of patients with axSpA, distinguishing this disease from both healthy controls and CD, and pointing to a disease-specific alteration in gut-bone signaling. This disease specificity is underscored by the observation that inflamed ileum from CD patients does not display reduced sclerostin, highlighting unique disease mechanisms in axSpA. Consistent with our previous work on circulating sclerostin [10], intestinal sclerostin levels did not correlate with articular disease activity, suggesting that sclerostin reflects bone and mucosal processes rather than joint inflammation.

It might be speculated that sclerostin underproduction is unrelated to the degree of intestinal inflammation and represents a specific feature of SpA, mirroring a phenomenon that has been observed for other pathways, further distinguishing gut inflammation in IBD from subclinical intestinal inflammation in SpA [29]. Although both CD and axSpA are associated with gut dysbiosis, comparative studies demonstrate that their microbiomes are compositionally distinct [30], supporting engagement of different downstream pathways. This divergence likely explains why intestinal sclerostin is reduced in axSpA but not in CD. Recent data further suggest that in CD, fungal components of intestinal microbiota may directly sustain sclerostin production [31], a mechanism not described in SpA. Previous clinical studies investigating serum sclerostin in CD have similarly not consistently shown reductions, but somewhat mixed results [10, 31, 32]. Conversely, in IBD patients who also have concomitant SpA, serum sclerostin is lower and anti-sclerostin antibodies higher, supporting the idea that sclerostin loss is driven by SpA rather than intestinal inflammation per se [10].

Significantly, our study extends these observations by demonstrating, for the first time, that intestinal production of sclerostin follows the same pattern: preserved or increased in CD but markedly reduced in SpA, underscoring the disease specificity of this pathway. A key novel aspect of our study is the identification of a sclerostin-serotonin axis in the gut. We observed that reduced sclerostin expression in axSpA correlates with increased serotonin production. Mechanistically, sclerostin modulates serotonin synthesis by regulating TPH1, the rate-limiting enzyme in this pathway. While our data indicate that sclerostin downregulation is associated with increased TPH1 expression, it remains to be clarified whether this effect is direct at the transcriptional level or mediated by secondary factors. Our data suggest that sclerostin counteracts WNT signalling, thereby controlling serotonin production through regulation of TPH1 expression.

Notably, higher serotonin levels have also been reported in CD, but this appears to result from serotonin reuptake (SERT downregulation) rather than enhanced biosynthesis via TPH1 [33, 34]. By contrast, in SpA, we demonstrate a sclerostin-dependent upregulation of TPH1, linking reduced intestinal sclerostin to increased serotonin synthesis. This interpretation is further supported by multi-omics analyses showing that alterations in the tryptophan–serotonin pathway are specific to axSpA and CD + axSpA, but not to CD alone [35]. Thus, both diseases may feature elevated serotonin, but through distinct upstream mechanisms, reinforcing that the sclerostin–serotonin axis is specific to SpA. Patients with concomitant axSpA and IBD were excluded to avoid confounding, and only a small CD cohort was included as a comparator for the inflamed ileum. Therefore, we did not investigate the condition of patients with both axSpA and CD in this study.

Locally, elevated intestinal serotonin can inhibit autophagy via mTOR, promoting dysbiosis and colitis, and similar effects have been shown in human immune cells [36]. Autophagy, in turn, regulates mucosal IL-23 expression in axSpA [37]. Thus, reduced intestinal sclerostin, accompanied by enhanced serotonin synthesis, may also contribute to axSpA by disrupting autophagy-dependent immune regulation, providing a plausible mechanistic link between serotonin metabolism, gut inflammation, and systemic disease.

The interpretation of serotonin measurements in SpA is complicated by both conflicting literature and significant methodological challenges. Previous studies have reported contrasting results: some found decreased serotonin in ankylosing spondylitis and further reductions during biologic treatment, while others observed no significant changes or a predominance of the kynurenine pathway over serotonin metabolism [38, 39]. Conversely, our data are consistent with findings from a recent study by Guła et al. (2024), which used LC-MS to demonstrate significantly higher circulating serotonin levels in patients with axial spondyloarthritis compared to healthy controls [40].

These discrepancies may reflect not only biological heterogeneity but also differences in assay modality and normalization strategies [41–43]. Peripheral serotonin synthesized by EC cells is actively taken up by platelets via the serotonin reuptake transporter (SERT) and sequestered into dense granules through vesicular monoamine transporter 2 (VMAT-2) [23]. In our study, only the measurement of intracellular platelet serotonin content revealed significant differences between groups, whereas ELISA did not (data not shown). This suggests that the choice of assay can critically affect the detection of disease-related changes. Moreover, since circulating serotonin is bound to platelet abundance, normalization for platelet count is essential to avoid confounding, and our findings indicate that intracellular platelet serotonin may better reflect disease-related alterations than extracellular measurements.

For the platelet serotonin assay, additional biological confounders—including circadian variation, sex differences, dietary intake, and medication use—were carefully controlled: we excluded individuals taking serotonin transporter inhibitors (SSRIs) or nutritional supplements, all donors were non-smokers, and blood samples were collected in matched pairs at the same time of day. While all participants followed a standard Mediterranean diet without specific restrictions, detailed dietary adherence was not assessed. Future studies should address these variables more systematically, and we strongly recommend the adoption of standardized, multimodal measurement protocols to improve reproducibility and comparability across studies.

Our findings in animals also suggest that gut microbiota composition is a significant determinant of intestinal sclerostin levels. The restoration of sclerostin following antibiotic modulation of the microbiota in HLA-B27 transgenic rats, along with the lack of serotonin reduction in germ-free conditions, supports the hypothesis that microbial factors contribute to the gut-bone axis in this disease [4, 44]. This finding is consistent with evidence that gut microbiota can influence host tryptophan metabolism, modulating both the serotonin and kynurenine pathways, and that variability in microbiota composition may help explain some of the contradictory results observed in the literature [45] In addition, it suggests a possible contribution of HLA-B27, known also to contribute to dysbiosis in human and animal models [46, 47]. The axSpA patients enrolled in this study were all HLA-B27 carriers; although our healthy controls were not HLA-B27 typed, the prevalence of HLA-B27 in the Italian population is lower (5–8%) [48].

We further demonstrated LPS-induced upregulation of serotonin receptors such as HTR2B in entheseal stromal cells, suggesting a mechanistic link between microbial products, serotonin signaling, and the pathogenesis of entheseal inflammation and new bone formation in axSpA. Specifically, circulating levels of lipopolysaccharide (LPS)—a component of Gram-negative bacterial cell walls—are elevated in axSpA, likely reflecting increased intestinal permeability and dysbiosis-associated microbial translocation [49]. Once in circulation, LPS may reach peripheral musculoskeletal sites, including the enthesis, where it can activate innate immune pathways and modulate local cell phenotypes. Our data suggest that LPS directly induces HTR2B expression in entheseal cells, thereby enhancing their responsiveness to serotonin. This upregulation may promote pro-osteogenic signaling in the context of chronic inflammation, providing a functional link between gut-derived microbial cues, serotonergic signaling, and pathological bone remodeling in axSpA [50–52].

While this pathway deserves further exploration, alternative mechanisms may contribute to explaining the effect of sclerostin deficiency on arthritis. Sclerostin deficiency has also been shown to contribute to enhanced Th17 responses and increased inflammation in experimental arthritis models, supporting the immunomodulatory role of sclerostin beyond bone metabolism [53]. In this regard, dendritic cells (DCs) lacking LRP5/6-β-catenin signaling have been shown to display an increased production of pro-inflammatory cytokines and a decreased production of anti-inflammatory cytokines such as IL-10 and IL-27 [54].

While our study provides new insights into the role of sclerostin in the gut-bone-immune axis, several limitations must be considered. The cross-sectional design precludes causal inference, and although our sample size was sufficient to detect significant differences, it may not capture more subtle associations. Platelet serotonin measurements were restricted to a small, freshly recruited subset of patients and controls, selected to minimize confounding factors; nevertheless, the difference between groups was evident despite the limited sample size, supporting the robustness of this finding.

The lack of longitudinal data means we cannot determine whether restoration of intestinal sclerostin precedes clinical improvement or altered disease progression. It will be essential for future research to incorporate serial sampling and interventional trials, for example, by testing whether treatment leads to the restoration of intestinal sclerostin and its impact on serotonin levels, clinical improvement, or radiographic progression. Additionally, the interpretation of serotonin data is complicated by both biological variability and methodological challenges, as discussed above [38].

In summary, our findings identify intestinal sclerostin downregulation and altered serotonin pathways as novel features of axSpA, linking gut dysbiosis and bone pathology. These results expand the biological repertoire of sclerostin and suggest potential therapeutic targets within the gut-bone axis. Future work should prioritize longitudinal sampling and interventional trials to clarify causality and therapeutic potential.

## Supplementary Information

Below is the link to the electronic supplementary material.

Supplementary Material 1 (DOCX 24.0 KB)

Supplementary Material 2 (DOCX 20.0 KB)

## Data Availability

Datasets are available on reasonable request to the corresponding author.

## Declarations

### Declarations of generative AI and AI-assisted technologies in the writing process.

During the preparation of this work, the authors utilized ChatGPT-4 (OpenAI) to enhance readability and language clarity. The authors reviewed and edited all AI-generated content as needed and take full responsibility for the content of the published article.
