# Supplementary material for: Intestinal Sclerostin Deficiency Links Gut Dysbiosis to Altered Serotonin Homeostasis in Axial Spondyloarthritis
Source: Inflammation. 2026 Jan 10;49(1):38. doi: 10.1007/s10753-025-02393-3 (PMC12858532; doi:10.1007/s10753-025-02393-3)
Supplement: Supplementary file 1 — Supplementary Material 1 (DOCX 24.0 KB) [file 10753_2025_2393_MOESM1_ESM.docx]

**Supplemental Table 1. Characteristics of patients and controls**

|  | **axSpA (N=25)** | **Controls (N=20)** | **CD**  **(N=6)** | **Sig.** |
| --- | --- | --- | --- | --- |
| **Age, mean (range) years** | 38 (22-­‐55) | 42 (21-­‐60) | 29 (22-45) | *ns* |
| **Sex, no. (%) female** | 2 (8) | 1 (5) | 1 (17) | *ns* |
| **HLA-­‐B27 (%)** | 100 | -*na* | *na* | -­‐ |
| **Obese n (%)** | 4(16) | 2 (10) | 0 (0) | *ns* |
| **Disease duration from diagnosis, mean (range) months** | 22 (11-­‐44) | -­‐ | -­‐ | -­‐ |
| **CRP (mg/l), mean (range)** | 3.6 (1.5-­‐6.8) | -­‐ | -­‐ | -­‐ |
| **Axial involvement, no. (%)** | 25 (100) | -­‐ | -­‐ | -­‐ |
| **Peripheral arthritis, no. (%)** | 4 (16) | -­‐ | -­‐ | -­‐ |
| **Enthesitis/dactylitis, no. (%)** | 2 (8) | -­‐ | -­‐ | -­‐ |
| **Uveitis, no. (%)** | 1 (4) | -­‐ | -­‐ | -­‐ |
| **ASDAS score, mean (range)** | 3.8 (2.6-­‐5.2) | -­‐ | -­‐ | -­‐ |
| **Crohn's Disease Activity Index (CDAI), mean (range)** | -­‐ | -­‐ | 255,2(157-­‐346) |  |
| **NSAIDs (%)** | 4(16) | -­‐ | -­‐ | -­‐ |
| **Salazopyrin (%)** | 0 | 0 | 0 | -­‐ |
| **Methotrexate (%)** | 0 | 0 | ‐0 | -­‐ |
| **TNF-­‐blocking agents (%)** | 0 | 0 | 0 | -­‐ |
| **IL-­‐17 inhibitors** | 0 | 0 | 0 | -­‐ |

p values for age were calculated using the Kruskal–Wallis test; p values for sex were calculated using Fisher’s exact test. Abbreviations: AxSpA, axial spondyloarthritis; HC, healthy controls; CD, Crohn’s disease; HLA, human leukocyte antigen; CRP, C-reactive protein; ASDAS, Ankylosing Spondylitis Disease Activity Score; CDAI, Crohn’s Disease Activity Index; NSAIDs, non-steroidal anti-inflammatory drugs; TNF, tumor necrosis factor; IL, interleukin.
