# Supplementary material for: Intestinal Sclerostin Deficiency Links Gut Dysbiosis to Altered Serotonin Homeostasis in Axial Spondyloarthritis
Source: Inflammation. 2026 Jan 10;49(1):38. doi: 10.1007/s10753-025-02393-3 (PMC12858532; doi:10.1007/s10753-025-02393-3)
Supplement: Supplementary file 2 — Supplementary Material 2 (DOCX 20.0 KB) [file 10753_2025_2393_MOESM2_ESM.docx]

**Supplemental Table 2: List of reagents**

| **Target** | **Antibody** |
| --- | --- |
| Anti-sclerostin antibody | s5545, Sigma Aldrich |
| TPH1 | ab52954 (Abcam) |
| GAPDH | 14C10, Cell Signaling |

| **Recombinant protein** |  |
| --- | --- |
| Human Sclerostin | 100-49-20UG, Peprotech |
| Human WNT3a | P9412, Abnova |

| **Primers** | |
| --- | --- |
| TPH1 mouse | Hs00188220_m1 |
| GAPDH | Hs02786624_g1 |
| 18S | Hs03003631_g1 |
| TPH1 human | Fwd: ACGTCGAAAGTATTTTGCGGA  Ref: ACGGTTCCCCAGGTCTTAATC |
| Sclerostin Human | Fwd: ACACAGCCTTCCGTGTAGTG  Red: GGTTCATGGTCTTGTTGTTCTCC |

| **ELISA Kit** | |
| --- | --- |
| Serotonin | ab133053 (Abcam) |
